# Retinoblastoma Makes Its Mark on Imprinting in Plants

**DOI:** 10.1371/journal.pbio.0060212

**Published:** 2008-08-26

**Authors:** Liliana M Costa, José F Gutierrez-Marcos

## Abstract

Imprinting in flowering plants and mammals causes monoallelic expression of parental alleles, but how is this achieved? New evidence in plants implicates the retinoblastoma pathway in establishing imprints during gametogenesis.

Genomic imprinting results in the preferential expression of alleles from either the maternal or paternal chromosomes. This epigenetic process occurs in embryonic and extra-embryonic (placental) tissues of mammals, but only in the extra-embryonic tissue (the endosperm) of flowering plant seeds. It is thought that imprinting arose to ensure that both maternal and paternal genomes contribute to the development of the offspring [1]. Indeed, the predominant occurrence of imprinting in extra-embryonic structures might indicate a similar reproductive strategy in both taxa to correctly regulate embryo growth and development. Because extra-embryonic lineages do not contribute any genetic information to the offspring, it is likely that epigenetic mechanisms responsible for imprinting could evolve rapidly [2,3], without causing deleterious consequences to the offspring.

Genomic imprinting was first recognized in maize through genetic studies revealing non-Mendelian maternal expression of a seed pigmentation gene [4]. Almost 40 years later, relatively few imprinted genes have been identified in plants, compared with over 80 genes that are imprinted in mammals [5]. Through analysis of some of these genes, we are beginning to understand the important roles played by certain epigenetic factors, especially those involved in DNA and histone methylation, in the regulation of genomic imprinting. Despite these advances, the precise molecular basis for the establishment of imprints is far from clear.

Most known mammalian imprints undergo a cycle of germline establishment, somatic maintenance, and imprint erasure. Imprints are erased in the early germline and new imprints become established at late stages of gametogenesis by DNA methylation at imprinted control regions (ICRs) [6] through action of Dnmt3a and Dnmt3l DNA methyltransferases [7]. In addition, differential methylation of imprinted loci in male and female gametes is mediated by proteins that bind to individual demethylated ICRs [8] and act as insulators to protect them from remethylation. The epigenetic marks established on ICRs are maintained throughout development by the Dnmt1 DNA methyltransferase. These DNA methylation marks recruit histone modifying enzymes, such as embryonic ectoderm development (Eed), which is a member of the Polycomb group (PcG) protein complex [9]. The PcG complex acts to maintain transcriptional silent states of some imprinted genes by introducing methylation marks to histone-3 tails at these (silent) loci [10].

As in mammals, imprinted loci in plants maintain their transcriptional silent states through the acquisition of epigenetic marks. These marks may include histone modifications by PcG complexes, or DNA methylation by the DNA methyltransferase enzyme MET1, a homolog of mammalian Dnmt1. Current data suggest that imprints are set during gametogenesis. In plants, female gametogenesis results in the formation of two gametes: the haploid egg cell and the homodiploid central cell, which contains two haploid nuclei, while male gametogenesis results in the formation of two genetically identical haploid sperm cells. In most cases, methylation is thought to be the primary epigenetic state of imprinted genes in the gametes. During gamete differentiation, demethylation is known to occur at some imprinted loci in the central cell, thus establishing differentially methylated regions (DMRs) between male and female gametes [11–14]. DEMETER (DME), a DNA glycosylase involved in DNA base-excision repair [15], has been shown to be responsible for DNA demethylation at some imprinted loci. However, it is likely that additional factors (possibly including other DNA excision repair enzymes) are also involved in the demethylation of imprints.

In mammals, loss of methylation is believed to occur through active and passive processes, either by base-excision repair or by replicating DNA in the absence of DNA methyltransferases, respectively [16]. In support of the latter, recent reports showed that the mammalian cell cycle regulator protein Retinoblastoma (RB) is involved in the transcriptional repression of *Dnmt1* [17,18]. RB functions in a complex with other RB-binding proteins, including histone deacetylases (HDACs) and the mammalian homologs of the plant PcG WD-40 protein MULTICOPY SUPPRESSOR OF IRA1 (MSI1) (RbAp46/48), to regulate transcription of E2F target genes. This offers a tantalizing new model for the regulation of DNA methyltransferases in a cell-cycle dependent manner. Given the remarkable similarities in the recruitment of specific epigenetic machinery by mammals and plants, the question is: do plant imprints also passively lose methylation during the cell cycle through a similar process?

Until now, Arabidopsis RB and its associated WD-40 protein, MSI1, have only been implicated in the regulation of female gametophyte (embryo sac) and early seed development [19–22]. It was not known if the RB-MSI1 complex was also required for imprinting. Interestingly, mutations in imprinted PcG genes *MEDEA (MEA)* and *FERTILIZATION INDEPENDENT SEED 2 (FIS2)* give rise to overproliferation phenotypes similar to those exhibited by *rb* and *msi* mutants, hinting that the RB-MSI1 complex might be controlled by components of the PcG pathway and/or that it might also regulate imprinting (Figure 1). In this issue of *PLoS Biology*, Pauline Jullien and colleagues attempt to explore the possible role of the cell-cycle-dependent RB-MSI1 pathway in regulating imprinting through repression of DNA methylation [23]. In their paper, the authors demonstrate transcriptional repression of *MET1* by RB and MSI1 during female gametogenesis. Direct binding of RB and MSI1 to the *MET1* promoter occurs at a potential E2F binding sequence, thus suggesting that the RB pathway in plants regulates DNA methyltransferase activity during the cell cycle, in a similar manner to mammals [24] (see Figure 1). It remains to be shown if *MET1* repression by RB also involves associated HDAC activity in plant female gametes. Interestingly, a subset of plant HDACs are able to physically interact with RB through amino acid residues other than the typical LxCxE motif [25], and are required for correct reproductive development in Arabidopsis [26], suggesting a degree of functional overlap with the RB-MSI1 complex. Thus a difficult task ahead would be to demonstrate if and how these protein complexes associate in vivo during female gametogenesis to repress *MET1* transcription. Another important issue to resolve is whether the target specificity of the RB-MSI1 complex is determined by E2F transcription factors, or if the complex targets *MET1* alone in a context-specific manner.

**Figure 1:**
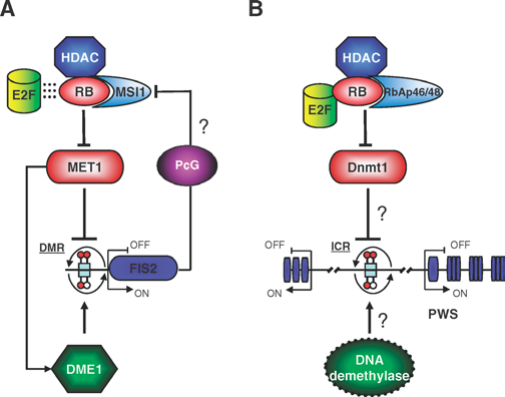
Establishment of Epigenetic Marks on Imprinted Genes During Gametogenesis The expression or repression of imprinted genes in plants and mammals is mediated by the differential methylation of regulatory regions. (A) In plants, during female gametogenesis, the RB-MSI1 complex may interact with E2F transcription factors (dotted lines) and HDACs to repress the expression of the DNA methyltransferase *MET1*. MET1 protein acts at DMRs (light blue rectangles with lollipops) of imprinted genes, such as *FIS2*, to deposit a methylation mark (red filled lollipops), resulting in transcriptional inactivation. This epigenetic mark can be removed (empty lollipops) in the central cell, by a base-excision/mismatch repair DNA glycosylase enzyme (DME1), thus leading to transcriptional activation. It is likely that *DME1* transcriptional activity in the central cell is positively regulated by MET1, and that transcriptional activity of the RB/MSI1 complex might be regulated by PcG proteins, hence resulting in a two-step feedback loop regulation. (B) In mammals, during oogenesis, the RB/RbAp/HDAC complex interacts with E2F transcription factors to repress the transcriptional activity of the DNA methyltransferase *Dnmt1* gene. Dnmt1 targets ICRs (light blue rectangles) in the genome to maintain methylation marks (red filled lollipops) important for the long-distance sex-specific transcriptional regulation of imprinted genes, such as the PWS gene cluster. It is likely that either before fertilization or in the zygote, methylation marks in ICRs are actively removed (empty lollipops) by demethylation enzymes.

In the absence of functional RB or MSI1, *MET1* is up-regulated and expressed in the mature embryo sac, including in both female gametes. As a result, maternal expression of some but not all imprints is affected. This suggests that RB-MSI1 regulates a subset of imprinted genes, reinforcing the notion that distinct mechanisms are operating to establish monoallelic expression in plants [11]. Future research will no doubt focus on understanding the significance of *MET1* transcriptional repression prior to maturation of the wild-type embryo sac and differentiation of the female gametes. In particular, it will be important to compare MET1 protein levels and determine the degree of methylation at different imprinted loci in the gametes. These studies will be central to uncovering whether the maternal MET1 protein threshold is sufficient to maintain cytosine methylation at specific loci equally in both gametes. Further studies are also required to better understand the complex interplay between DNA methylation and demethylation in the gametes, especially in light of recent evidence indicating that cytosine methylation by MET1 positively regulates *DME1* expression [27]. In particular, it will be fascinating to determine if DME enzyme activity correlates with an elevated level of residual MET1 protein activity in the central cell compared with the egg cell, due to dosage derived from its homodiploid genomic constitution. It may simply be a case of correct imprinting establishment requiring a fine balance between DNA methylation and demethylation activities; a process that is coordinated by the RB-MSI1 pathway through controlled gene expression of *MET1*, and perhaps *DME1,* in a cell-cycle-dependent manner (Figure 1).

Thus the Retinoblastoma pathway has emerged as an important regulatory component in the establishment of genomic imprinting in plants. The results obtained by Jullien et al. and the conservation of the transcriptional repression of *MET1/Dnmt1* by the retinoblastoma pathway point to the potential involvement of RB in the control of imprinting in mammals (see Figure 1). In support of this hypothesis, it was reported that the imprinting of genes associated with Prader-Willi syndrome (PWS) is regulated by RB-binding proteins [28]. As with most significant findings, the discovery of the role of RB in imprinting regulation raises many intriguing questions and paves the way for a whole new exciting avenue of investigation. Indeed, the next challenging task ahead will be to ascertain how the RB complex itself is regulated to coordinate methylation events in the gametes. The discovery of the mechanism(s) regulating RB complexes in plants and mammals is certain to provide greater understanding about the molecular events leading to the establishment of imprinting marks.

## Abbreviations

DME: DEMETER
DMR: differentially methylated region
*FIS2*: *FERTILIZATION INDEPENDENT SEED 2*
HDAC: histone deacetylase
ICR: imprinted control region
MSI1: MULTICOPY SUPPRESSOR OF IRA1
PcG: Polycomb group
PWS: Prader-Willi syndrome
RB: Retinoblastoma
